# An Analysis of the Nutritional and Health Values of *Caulerpa racemosa* (Forsskål) and *Ulva fasciata* (Delile)—Two Chlorophyta Collected from the Philippines

**DOI:** 10.3390/molecules25122901

**Published:** 2020-06-24

**Authors:** Rexie P. Magdugo, Nolwenn Terme, Marie Lang, Hugo Pliego-Cortés, Christel Marty, Anicia Q. Hurtado, Gilles Bedoux, Nathalie Bourgougnon

**Affiliations:** 1 Laboratoire de Biotechnologie et Chimie Marines, EA3884, UBS, IUEM, F-56000 Vannes, France; magdugo@univ-ubs.fr (R.P.M.); nolwenn.terme@univ-ubs.fr (N.T.); marie.lang@univ-ubs.fr (M.L.); hugopliegocortes@gmail.com (H.P.-C.); christel.marty@univ-ubs.fr (C.M.); gilles.bedoux@univ-ubs.fr (G.B.); 2 Integrated Services for the Development of Aquaculture and Fisheries (ISDA) Inc., MacArthur Highway, Tabuc Suba, Jaro, Iloilo City 5000, Philippines; anicia.hurtado@gmail.com

**Keywords:** human food, amino acids, lipids, green seaweeds, biological activities

## Abstract

Polysaccharides, lipids and amino acid profiles were investigated to understand the nutritional value of *Caulerpa racemosa* and *Ulva* *fasciata* from the Philippines. The results revealed that both species contain high amounts of proteins (8.8–19.9% for *C. racemosa* and 8.0–11.1% for *U.* *fasciata*). The portions of the total amino acids that were essential amino acids (EAAs) (45.28 ± 0.12% for *C. racemosa* and 42.17 ± 0.12% for *U. fasciata*) out were comparable to FAO/WHO requirements. Leucine, valine, isoleucine, and lysine are the dominant EAAs in *C. racemosa*, while leucine, valine, lysine, and phenylalanine are those in *U. fasciata*. The fatty acid profiles are dominated by monounsaturated fatty acids and polyunsaturated fatty acids in *C. racemosa* (56.2%), while saturated fatty acids (72.1%) are dominant in *U. fasciata*. High C18/C20 polyunsaturated fatty acid ratios were recorded in both species. Mineral contents for both seaweeds were within levels considered safe for functional foods. Total pigment content of *C. racemosa* (140.84 mg/g dw) was almost 20 times higher than that of *U. fasciata* (7.54 mg/g dw). Hot water extract (HWE) from *C. racemosa* showed in vitro antiherpetic activity without cytotoxicity. Nutritional characteristics confirmed that *C. racemosa* could be potentially used as a nutritious and functional food items for human consumption.

## 1. Introduction

Many years ago, the Greek physician Hippocrates emphasized the importance of food and good health [1]. Even today, foods satisfy hunger, provide necessary nutrients, improve the physical and mental welfare of consumers, and prevent nutrition-related diseases like cardiovascular disease and osteoporosis [2,3]. Population growth drives the need to find new protein, fiber, and mineral sources to feed future populations and fight food insecurity. Insects and marine algae have been proposed as novel and sustainable foods [4]. Marine macroalgae contain significant quantities of not only vitamins, minerals, dietary fibers, proteins, and polysaccharides, but also various functional polyphenols, antioxidants, pigments, and lipids [2,5,6]. Biochemical contents can vary with species, geographical location, season, and temperature. Marine macroalgae exhibit a wide diversity of minerals and trace elements, such as sodium, calcium, chlorine, magnesium, zinc, and copper. Algal protein content is generally high; this is especially the case for red algae, in which protein content represents 40% of the dry matter [7,8,9]. Furthermore, the storage polysaccharides (starch, laminarin, floridoside) are diverse. Cell walls present low cellulose and hemicellulose contents and original phycocolloids (alginates, carrageenan, agars). Lipids (more particularly, polyunsaturated fatty acids), sterols, and vitamins such as vitamins C or B12 with antioxidant properties are also present in macroalgae. The search for new food trends is expected to increase in the future and encourage the consumption of algae. These trends include eating less meat, eating vegan products (in favor of vegetable proteins), growing concerns about all aspects of sustainable and clean food production, and "snacking". With these changes in attitude, the integration of macroalgae as an alternative source in the daily diet has immense potential. 

The Philippines is known for its rich flora, and its marine algae are a significant and diversified form of natural crop production. There are 1291 taxa of marine macrobenthic algae [10], and many species (350) are of economic importance as food and sources of industrial products such as polysaccharides, bioactive and natural nutritional products, and growth-promoting substances. The Seaweed Industry Association of The Philippines (SIAP) reported that the total production of all types of dried seaweeds in the Philippines from 2011–2015 was an average of almost 88,000 metric tons per annum. The local price of macroalgae ranged from $0.45 to $0.89 per kilogram throughout the year, according to sources in significant cities such as Tawi-Tawi, Zamboanga, Palawan, Cebu, and Manila [11]. The local price of macroalgae is considered affordable to average Filipinos, especially for those who live in big cities. Farming of *Caulerpa* and *Ulva* is expanding worldwide [5,11,12,13] to meet this growing demand from the health food market. In the Philippines, edible *Caulerpa* spp. (e.g., *C. lentillifera*, *C. peltata*, *C. racemosa*, *C. sertularioides*, and *C. taxifolia*) [5] were cultivated in sufficient amounts with *Kappaphycus* sp. and *Eucheuma* sp. [11], while *Ulva* spp. was planned to be cultivated along with *Halymenia* spp., *Sargassum* spp., and *Pyropia* spp. [11]. Following the prohibition of cultivation in mangrove areas, the production of *Caulerpa* declined. However, promising studies have recently been conducted in open-sea cultivation [14]. The *Ulva* spp. are encouraged by the Philippine government to be developed as a potential human and animal food, as well as a biogas feedstock. Several *Ulva* species are listed: *U. lactuca*, *U. rigida*, and *U. fasciata*. Meanwhile, *Caulerpa* consists mainly of two species, *C. lentillifera* and *C. racemosa*, which are the third most cultivated macroalgae in many parts of East Asia [15]. Consumers highly appreciate their delicate flavor and crisp texture, so these species are called "Green Caviar." Both *Caulerpa* and *Ulva* present many uses as human or animal food, and they are a source of minerals such as calcium, potassium, magnesium, sodium, copper, iron, zinc, and vitamin E. Culinary preparation varies according to species and country. Seaweeds can be consumed dry, fresh, boiled, in salads, as condiments, or cooked [16]. Some biological activities, such as antifungal activities, lowering blood pressure, or anthelmintic activities, are also mentioned [17,18,19]. *Ulva* and *Caulerpa* species have long been listed in the FAO database as two of the main macroalgae for commercial use and especially as direct food for human beings in various parts of the world [20]. Both green seaweeds constitute promising candidates as functional foods naturally enriched in nutrients and various health-promoting compounds. In the Philippines, these species of green seaweeds offer social and economic benefits and have a high level of consumer acceptance [16]. *C. racemosa* could be packed fresh in baskets lined with banana leaves or preserved in brine-cured or salted form [21]. *Ulva* is used in the form of fresh salad or as an ingredient in various food preparations [22]. In aquaculture, *Ulva lactuca* and *U. rigida* are also used as an alternative source of nutrients for aquafeeds, replacing soybean meal in the diet of shrimp [23]. Despite this information, little is known about the nutritional properties or biological activities of compounds extracted from *Caulerpa racemosa* and *Ulva fasciata*. De Gaillande et al. [5] presented a review in 2017 about the consumption, nutritional value, and farming of *Caulerpa* in the Indo-Pacific region, describing a genus rich in proteins, fibers, minerals, vitamins, polyunsaturated fatty acids, and bioactive antioxidants. 

Little is known about the nutritional properties or biological activities [24,25] of the *Caulerpa* and *Ulva* species from the Philippines. This study was conducted to give additional information on the potential of wild *Caulerpa* and *Ulva* species from this country. The present study therefore deals with the clarification of the fatty acid composition in relation to total lipids, along with the characterization of cell wall polysaccharides, proteins, amino acids, mineral compositions, and nutritional values. Furthermore, this study evaluates of the antiviral activity of parietal polysaccharides and the antioxidant activities of the different fractions in the context of health.

## 2. Results

### 2.1. Biochemical Composition Analysis of Raw Seaweeds and Their Extracts

The biochemical composition of the two green seaweeds, the hot water extract from *Caulerpa racemosa* and the crude ulvan from *Ulva fasciata,* are shown in Table 1. *C. racemosa* as raw seaweed presented high neutral sugar content (31.2%), followed by ash (29.4%), protein (19.9%), and sulfate group (13.0%) contents. The biochemical analysis of raw seaweed from *U. fasciata* indicated a composition rich in sulfate groups (25.4%), followed by total sugars (25.0%), ash (23.3%), and uronic acids (20.7%). Protein content constituted a little over 1/10 (11.1%) of the dry matter. Carbohydrate contents, including sulfated polysaccharides (corresponding to uronic acids, sulfate groups, and neutral sugars) in *C. racemosa* and *U. fasciata*, were about 47.8% and 71.1%, respectively. For *C. racemosa*, the hot water extract (HWE) with 13% yield presented neutral sugars (16.6%), sulfates (10.5%), and proteins (8.8%). Finally, the *U. fasciata* crude ulvan polysaccharide yield represented 23.71%, with major components including uronic acids (10%), neutral sugars (9.5%), and proteins (8.0%). 

### 2.2. Fatty Acid Methyl Ester (FAME) Analysis

The fatty acid (FA) content of *C. racemosa* represents 10.3% of the total lipid (TL) fraction and therefore 463 mg/100 g of dry seaweed. For *U. fasciata*, the FA content is 10.7% of TLs and therefore represents 439 mg/100 g of dry seaweed. The FA compositions of *C. racemosa* and *U. fasciata* are shown in Table 2. Unsaturated fatty acids (monounsaturated fatty acids (MUFA) and polyunsaturated fatty Acids (PUFA)) were the primary fatty acids in *C. racemosa* (56.2% of total fatty acids (% TFAs)). In contrast, in *U. fasciata*, saturated fatty acids (SFAs) were the primary fatty acids (72.1% of TFAs). Unsaturated fatty acids from *C. racemosa* represented a variety of structures, with 14 different FAs identified by GC ranging from 0.4% to 28.1% of total FAs. The main FA detected was oleic acid (C18:1ω9, 28.1% of TFAs). In *U. fasciata*, palmitic acid was the most abundant FA, accounting for 59.9% TFAs, which amounts to 83% of the SFAs. There was less diversity in unsaturated FA, with six different MUFAs and five different PUFAs in *U. fasciata* compared to *C. racemosa*. The MUFA and PUFA contents ranged between <0.1 and 5.9% and between <0.1 and 5.3% of TFAs, respectively.

The nutritional quality indexes calculated with the fatty acid profiles of *C. racemosa* and *U. fasciata* are presented in Table 3. High C18/C20 PUFA ratios were recorded in both species. The PUFA/SFA ratio of *C. racemosa* was 0.52, whereas it was 0.15 for *U. fasciata*. Ratios based on the functional effects of FA were also calculated. For health, high fatty acid hypocholesterolemic/hypercholesterolemic (h/H) ratios are considered more beneficial as these are directly related to high PUFA content [26]. The atherogenic index (AI) and thrombogenic index (TI) are related to the protection against coronary artery diseases. The lower the value, the higher the protection properties [27]. *C. racemosa* exhibited lower AI and TI and higher h/H than *U. fasciata*.

### 2.3. Monosaccharide Composition Analysis

The unit sugar composition of purified fractions of *C. racemosa* and ulvans of *U. fasciata* was determined by high-performance anion-exchange chromatography (HPAEC). The results were expressed as a percentage of sugars relative to the total monosaccharides detected. Eleven monosaccharides were identified in the HWE fraction from *C. racemosa*. The main contents were glucose (52.4%), galactose (15.6%), mannose (12.6%), and xylose (8.2%); lower amounts of fructose (4.0%), glucuronic acid (1.4%), fucose (1.3%), and rhamnose (1.0%) were found; and there were trace amounts of arabinose, glucosamine, and glucoheptose. Meanwhile, there were seven monosaccharides identified in crude ulvan extracted from *Ulva fasciata*, characterized by high amounts of rhamnose (54%) and glucose (26.6%) and lower amounts of xylose (7.8%) and glucuronic acid (1.3%). The total monosaccharide contents were 198.13 ± 8.23 μg mg^−1^ of dw for HWE and 62.27 ± 1.6 μg mg^−1^ of dw for crude ulvan (Table 4).

### 2.4. Fourier-Transform Infrared (FTIR) Spectroscopy

**Figure 1:**
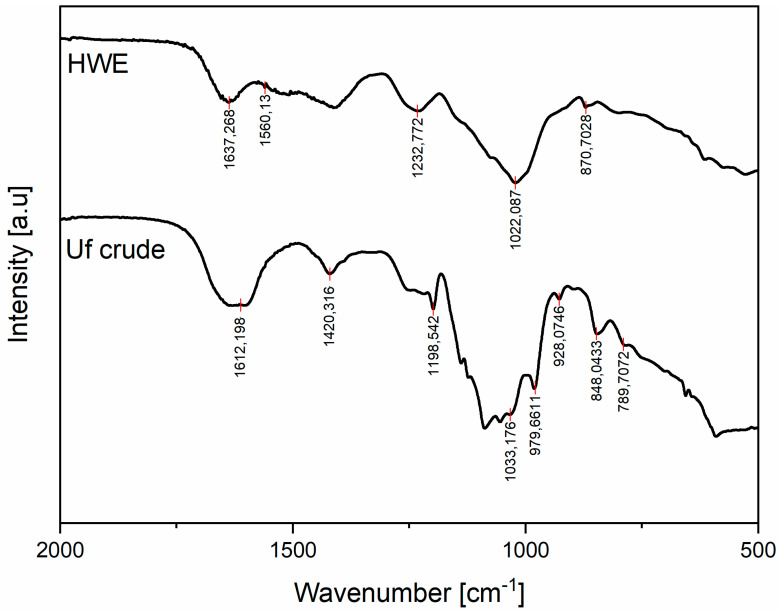
FTIR spectra of *C. racemosa* (HWE) and *U. fasciata* (crude ulvan) fractions.

The FTIR spectra and spectral bands of interest of *C. racemosa* (HWE) and crude ulvan from *U. fasciata* are shown in Figure 1. 

The IR spectrum of HWE and crude ulvan within the 2000–500 cm^−1^ zone or the fingerprint region for polysaccharides were used for data analysis. HWE showed spectral bands of high intensity at 1232 and 1022 cm^−1^, while bands of medium or low intensity were at 1637, 1560, and 871 cm^−1^. All spectral bands between 1260 and 1220 cm^−1^ related to the sulfation level in general [28,29]. The spectral bands at 1240–1230 cm^−1^ and 1022 cm^−1^ in HWE from *C. racemosa* were due to stretching of –SO_3_ and C-O in ring groups [30,31]. The medium and weak intensity bands at 1640–1630 cm^−1^ in HWE were due to carboxylic acid vibrations. The peaks at the 1561–1546 cm^−1^ bands in HWE indicate amide N-H bending vibrations [30]. The band at 871 cm^−1^ in HWE is characteristic of the presence of sulfate groups on the molecule [30,32,33].

Crude ulvan has specific spectral bands of high intensity at 1612, 1033, and 979 cm^−^^1^ and bands of medium or low intensity at 1420, 1198, 980, 928, 848, and 790 cm^−^^1^. 

Strong- and weak-intensity carboxylic vibrations were found around 1612 and 1420 cm^−1^. The strong intensity at around 1612 cm^−1^ corresponded to an asymmetrical stretching band attributed to the presence of OH groups, which is within the spectral bands of uronic acids [34]. The weaker intensity band at around 1420 cm^−1^ was symmetric stretching vibration of carboxylic groups [35]. Between 1200 and 1000 cm^−1^, the region was dominated by sugar ring vibrations that overlapped stretching vibrations of C-OH side groups and the C-O-C glycosidic bond vibrations. The double spectral band at 1198 cm^−1^ indicates the presence of sulfate groups that belongs to a band indicating S=O elongation bonding. The band at 1033 cm^−1^ had the highest intensity, corresponding to a C-O-S elongation in the equatorial position [36]. The bands around 980 cm^−1^ correspond to glycosidic linkages [36,37] or are associated with galactose-6-sulfate [37]. The bands around 848 cm^−1^ are characteristic of the presence of sulfate groups on the molecule.

### 2.5. Amino Acid Composition

The amino acid composition of the raw seaweeds *Caulerpa racemosa* and *Ulva fasciata* was established using gas chromatography and is presented in Table 5. The amino acid composition of ovalbumin, a reference protein from the egg white, is also shown in Table 5 for comparison. Amino acids have been classified according to their quality as essential (EAAs) or non-essential (NEAAs) amino acids. Leucine (9.64 ± 0.09%), valine (8.63 ± 0.02%), isoleucine (6.14 ± 0.10%), and lysine (5.96 ± 0.14%) are the most represented EAAs in *C. racemosa*. *U. fasciata* is rich in the EAAs leucine (9.31 ± 0.28%), valine (8.50 ± 0.07%), lysine (7.92 ± 0.44%), and phenylalanine (6.07 ± 0.35%). Both macroalgae are deficient in the EAAs histidine and methionine (<2.50% in *C. racemosa* and <1.5% in *U. fasciata*). High glutamic acid and aspartic acid (NEAAs) contents have been observed in both algae (18.70 ± 0.17% and 15.49 ± 0.14% glutamic acid content for *C. racemosa* and *U. fasciata*, respectively; 11.53 ± 0.11% and 10.95 ± 0.46% aspartic acid content for *C. racemosa* and *U. fasciata*, respectively). The EAA/NEAA ratio has been calculated for both algae. This value, which indicates of the quality of proteins, must tend towards or be greater than 1 in order to contribute to an optimal EAA intake. Observed EAA/NEAA ratios were 0.83 ± 0.00 in *C. racemosa*, and 0.73 ± 0.04 in *U. fasciata*.

### 2.6. Minerals

Major mineral elements (Ca, Mg, Na, and K) and trace elements (Fe, Zn, Cu, and Mn) were determined using a PerkinElmer AAnalyst 200 atomic absorption spectrophotometer (AAS) with a single hollow cathode lamp for each element and an air-acetylene burner.

The mineral contents expressed as the content of each mineral relative to the seaweed dry weight (% of dw) of *C. racemosa* and *U. fasciata* are given in Table 6. The percentages of the recommended daily intake covered by an intake of 8 g of dry seaweed are also presented, as is the daily recommended intake allowance [9]. *C. racemosa* had a high total macroelement content (16.83% dw); sodium had the highest content (7.48%), followed by potassium (5.30%), calcium (3.55%), and magnesium (0.5%). Microelement content was also high (1.03% of algal dw) and included iron (0.49%), zinc (0.42%), copper (0.11%), and manganese (0.006%). *U. fasciata* was rich in macroelements, presenting a total of 12.49% of algal dw; calcium had the highest content (4.80%), followed by sodium (4.71%), magnesium (2.24%), and potassium (1.51%). Microelements accounted for 2.10% of algal dw and were represented by iron (1.77%), zinc (0.27%), copper (0.06%), and manganese (0.003%). Eating 8 g of dry *C. racemosa* represents between 8.7% and 653.3% of the recommended daily intake for minerals. These percentages were high, especially for the microelements. For *U. fasciata*, 8 g of dry seaweed covered 2360% of the recommended daily intake (RDI) of iron. The Na/K ratios in *C. racemosa* and *U. fasciata* were 1.41 and 3.12, respectively.

### 2.7. Pigments

Pigment analysis was based on the method used by Silkina et al. [39] with some modifications. The analysis was performed by high-performance liquid chromatography (HPLC) on a Nucleodur C18 column (EC 250/4.6 100-5 C18ec Macherey-Nagel). A two-solvent elution (solvent A 100% 0–27 min, solvent B 100% 27–42 min) was used at a flow rate of 1.4 mL/min to separate the pigments. Pigment extraction from raw material (20 mg) was diluted with methanol (2 mL, at 4 °C) and was sonicated (pulse 2, amplitude 100) for 2 min in test tubes. Samples were kept in the darkroom (4 °C, 24 h), centrifuged (36,000× *g*, 4 min) and then filtered (0.22 µm, Minisart High Flow, Sartorius Stedim Biotech, Göttingen, Germany) before analysis.

Determinations of pigment composition are shown in Table 7. In this study, chlorophyll *a* and *b* and β-carotene were detected in both seaweeds. Chlorophyll *b* was the major pigment in both seaweeds, representing 81.42 ± 0.24 mg/g of dw for *C. racemosa* and 4.64 ± 0.60 mg/g of dw for *U. fasciata*. *C. racemosa* contained more pigments than *U. fasciata*. Total pigment content of *C. racemosa* (140.84 ± 2.33 mg/g dw) was almost 20 times higher than that of *U. fasciata* (7.54 ± 1.34 mg/g dw).

### 2.8. Evaluation of Cytotoxicity and Antiviral and Antioxidant Activities

After three days of treatment, microscopically visible alteration of normal cell morphology was observed, and the availability assay showed destruction of the cell layer. No cytotoxic effect of polysaccharides on the Vero cells was observed in the range of the concentrations assayed. No anti-HSV activity was detected for the crude ulvan. However, the hot water extract (HWE) fraction of *C. racemosa* showed antiherpetic activity with an EC_50_ of 85.59 ± 26.27 µg/mL (Table 8). Cellular protection of 70% was obtained for 200.0 µg/mL 72 h after infection. The free radical scavenging of the blank and the fractions of HWE and crude ulvan were estimated by the decrease in absorbance due to the reduction of the DPPH radical by the extracts. The BHA and BHT standards presented inhibiting concentrations (IC_50_) of 7.05 ± 0.59 and 5.32 ± 0.59 μg/mL, respectively. The HWE had 57.16% maximum scavenging activity at 5 mg/mL, while crude ulvan had 15.61% at 50 mg/mL. Samples of HWE and crude ulvan had weak antiradical effects in the concentration range used, with IC_50_ values of 276.08 and 4482.49 µg/mL, respectively. 

## 3. Discussion

The biochemical composition of *Caulerpa racemosa* collected in the Philippines agrees with previous studies of *Caulerpa* species in the Indo-Pacific regions [5,40]. In our study, *C. racemosa* protein content (19.9 ± 0.5% of dry weight) is within the concentration range (0.6–20.8%) of the majority of edible *Caulerpa* [5]. Osuna-Ruiz et al. [41] reported lower mean values of proteins (11.96%) and lipids (0.92%) from *C. sertularioides* collected in Sinaloa, Mexico. However, its reported ash content (35.54%) was higher than that found in the present study of *C. racemosa*. In terms of biochemical composition from polysaccharides extracts, Ji et al. [42] reported comparable protein contents (9.9%, 14.0%, and 2.0%) from *C. racemosa* extracted by neutral protease combined with hot water, compared to that of the HWE fraction (8.8%) of the present study. Meanwhile, the uronics content (3.9–7.9%) and sulfates content (27.6–48.3%) that they reported were higher than those of the HWE in the present study (1.6% and 10.54%, respectively). In general, total sugars were abundant components compared to proteins, phenols, and flavonoids [43], as was also observed for neutral sugars for HWE in the present study. On the contrary, another study by Hao et al. [43] on *C. racemosa* var. *peltata* polysaccharides, extracted by hot water at 85 °C for 3 h, reported lower protein (0.67%), polyphenols (0.19%), and total sugar (3.4%) contents [43] than the present HWE study. 

The biochemical composition of *Ulva fasciata* in the present study is in agreement with previous studies for other *Ulva* species [44,45,46]. Yedukondala Rao et al. [46] reported amounts of proteins (25.15%), carbohydrates (60.28%), and lipids (9.15%) that were comparable to those of *U. fasciata* in the present study. However, Yedukondala Rao et al. [46] reported lower ash content (5.41%) than that found in the present study of *U. fasciata*. Another study, considering tropical *U. expansa* from Sinaloa, Mexico, reported lower values of proteins (4.12%) and lipids (0.65%) but a higher value of ash (35.66%) [41] when compared to the present study of *U. fasciata*. A significant seasonal variation of biochemical composition in green algae has been previously reported concerning species and geographic areas [47,48]. *Ulva* spp. have high protein content, although it may vary from place to place (5–30% dw, according to Anh et al. [49]). For example, *Ulva pertusa* and *Ulva intestinalis* samples collected from Southern Thailand incorporate high contents of protein (14.6% and 19.5% of dw, respectively). Sulfates content (3.12%) in the present study is within the range of values published in the literature [35,50]. Protein content (8.0%) in the present study is much higher (by up to 20 times) than that found by Costa et al. [50] and is up to 2 times higher than that found by Hernández-Garibay et al. [35]. The uronic acid content (10.0%) in the present study is higher than that found by Hernández-Garibay et al. [35].

The total amino acid composition profiles of *C. racemosa* are in accordance with previously reported data [5]. High amounts of the non-essential amino acids (NEAAs) aspartic acid and glutamic acid are found in both algae, with 11.53 ± 0.11% and 18.70 ± 0.17%, respectively, in *C. racemosa* and 10.95 ± 0.46% and 15.49 ± 0.14%, respectively, in *U. fasciata*. This profile follows previously published data, as the two amino acids generally represent between 26% to 32% of the total amino acid content [51]. These two amino acids are generally found in large quantities in seaweeds and are responsible for their characteristic “umami” taste and sensation [52]. The EAA/NEAA ratio of 0.83 ± 0.00, which is in the range of previously reported data [5,43], indicates that *C. racemosa* is a balanced source of amino acids, close to ovalbumin (EAA/NEAA of 1.02). Given the total protein content and amino acid profile, this seaweed could be a good food resource to replace meat products. A lower EAA/NEAA ratio (0.73 ± 0.04) was observed for *U. fasciata*, which indicates that its consumption is less favorable for a good EAA intake. However, combining these two algae in the diet would lead to a complete supply of amino acids. The results concerning the content and the quality of the proteins in both algae must nevertheless be qualified, given the drying method used. It has been shown that solar and convective drying both involve in the destruction of amino acids in *Ulva* sp. (48.13 ± 2.15 and 41.38 ± 3.50 g EAA for 100 g of protein, respectively) when compared to vacuum drying (56.71 ± 1.91 g EAA for 100 g of protein) [53]. Therefore, the pretreatment of seaweeds should be carried out by other methods to preserve their quality.

Macroalgae typically do not exceed 2–4.5% of dw as lipids, mainly as phospholipids and glycolipids [18,54,55]. The lipid content of *Caulerpa* and *Ulva* species can be high (0.1–7.2% and 0.1–14.2% of dw, respectively), with a high content of PUFAs considered essential nutritional components in humans and animals. In the present study, we observed 4.5% of dw for *C. racemosa* and 4.1% of dw for *U. fasciata* in terms of lipids. The highest PUFA content was measured in *C. racemosa*, representing 60.8% of the total fatty acids. We observed a lower total PUFA content (21.6%), but this was comparable to the PUFA content in *C*. *racemosa var. laetevirens* [5]. The most important PUFAs are the essential fatty acids eicosapentaenoic acid (EPA; C20:5ɷ 3) and docosahexaenoic acid (DHA; C22:6ɷ 3) along with their precursors α-linolenic (ALA; C18:3ɷ 3) and linoleic acids (LA; C18:2ɷ 6). γ- and α-Linolenic (C18:3ω6 & ω3) and linoleic acids are the most abundant PUFAs in *C. racemosa* from the Philippines and are also present in *U. fasciata* according to the literature [5,41]. EPA, DHA, and arachidonic acid (C20:4ɷ 6) are also present in *C. racemosa*, as described previously for other *Caulerpa* species [5,41]. Polyunsaturated fatty acids and carotenoids are most noteworthy as functional foods [54,56]. These are reported to have several health benefits, such as the prevention of skin scaling, hair loss, cardiovascular diseases, and cancer [2,5,41,57]. The FAO/WHO considered that a PUFA/SFA ratio below 0.45 is undesirable, as the SFAs potentially increase the blood cholesterol level [58]. Our present results in *C. racemosa* show potential properties complying with consumption requirements. For health, high h/H ratios are considered more beneficial, as these are directly related to high PUFA content [26]. The results for *C. racemosa* agree with h/H values for marine fish like sardine or mackerel that are recognized as beneficial for health [59]. AI and TI are related to the protection against coronary artery diseases. The lower the value, the greater the protection properties [27]. The values of AI and TI for *C. racemosa* are in the range of reported *Caulerpa* species or lower than those of meat (1.08–1.58) and milk (2.1) [60]. Moreover, the ω6/ω3 ratio (<10) confirms the potential health benefits of the consumption of *C. racemosa* from the Philippines regarding the lipid distribution [61]. Concerning the nutritional quality indexes of fatty acids, *U. fasciata* cannot be defined as a potentially beneficial edible seaweed despite the presence of essential fatty acids. Indeed, the PUFA/SFA and h/H ratios are lower than the recommended values [58,59] for a beneficial assessment. Moreover, *U. fasciata* is rich in long-chain SFAs (i.e., myristic, palmitic, and stearic acids), which are thrombogenic and promote platelet aggregation [62]. Although the ω6/ω3 ratio for *U. fasciata* was >10, this value was attributed to PUFAs with C18 and not to the content of PUFAs with ≥C20. This *U. fasciata* species should still be considered a good-health promoter because of its considerable amount of C22:1ω9 (erucic) and C24:1ω9 (nervonic) acids, compounds which are said to prevent demyelinating disease [63].

Mineral content is one of the main nutritional characteristics of seaweeds taken up directly from their environment. Seaweeds absorb an incomparable wealth of mineral elements from the sea and are known as excellent sources of vitamins and minerals, especially potassium and iodine [19]. However, nutritional generalization of algal mineral content is difficult, mainly due to diversity arising from seasonality, geographic location, taxonomic variations, processing, and laboratory manipulations [18]. The total macroelement content of *C. racemosa* was higher (16.83%) than that of *U. fasciata* (12.49%) in the present study. Among the four macroelements tested, Na content was highest in both species, but the lowest was Mg in *C. racemosa* and K in *U. fasciata*. The differences in content are perhaps attributed to their morphological characteristics: *C. racemosa* is a stoloniferous plant with upright branches made up of ramuli and has higher capacity to absorb minerals in the surrounding water; in contrast, *U. fasciata* is a thin and membranous plant. Out of the microelements, Mn had the lowest content in both species, although in *C. racemosa* it was slightly higher.

Some macroelements of *C. racemosa*, such as Na and K, had higher values than those reported previously, while Ca and Mg were detected at similar levels compared to previous results in the same species [5,64]. The ratio of Na/K in *C. racemosa* from the Philippines was considered small (<1.5); thus, consumption of this seaweed can help balance high-Na/K-ratio diets that are common today [65]. However, Na/K ratios in the *Caulerpa* species were reported to be highly variable (0.3–2.3) depending on the methodology and the rinsing process [5]. The microelements in *C. racemosa* were all within the range of levels reported in previous studies [5,64,66]. Iron is essential for cell functions (oxygen and electron transport and DNA synthesis), while manganese helps in protein, lipid, and carbohydrate metabolism [67]. According to Garcia et al. [68], microelement concentrations vary with species, which could be due to morphological and physiological affinities for different metals. As the average daily consumption of dry seaweeds in Asia is 8 g and based on the results of the present study, the mineral contents of *C. racemosa* are within safe levels, giving it the potential to be a functional food for humans. For example, an 8 g portion of *C. racemosa* covered around 34% of the RDI of calcium, like other seaweeds, while the same quantity of cheddar cheese provides only 5% of the RDI [32,69]. In the Ulvophyceae, structurally diverse and heterogeneous sulfated polysaccharides [70] represent around 38–54% of dw. The present study showed that the ash content of the *U. fasciata* was 23.3% of dw, representing a high mineral content. The macroelements of *U. fasciata* obtained in the present study were found to be higher than those found in previous studies [64,66,68], with the exception of potassium, which was reported higher (3.77%) [68] in different *Ulva* species. The Na/K ratio in *U. fasciata* was 3.12, which was higher than previous results. The microelement contents were also found to be higher than previous results [68], except for manganese (0.048%) [66] and copper (0.071%) [68], which were higher in previous results. Results of the daily recommended intake allowance showed that the use of no more than 2.5 g of *U. fasciata* would not compromise health due to the iron content of this seaweed. The accumulation of excess iron may generate oxidative stress and increase cardiovascular risk. However, the biological availability of minerals is influenced by diet composition, due to synergistic and antagonistic interactions [71]. Moreover, only 1–53% of iron from food is absorbed by the human body [72].

The significance of determining the micro- and macroelemental contents of seaweeds is related to their consumption by humans. It is a known fact that Asian countries, primarily Japan, China, and Korea and to a lesser extent the Philippines, Indonesia, and Malaysia are seaweed-eating countries [65,73,74], and this has been associated with distinct health benefits, including cardioprotective, neuroprotective, and anti-inflammatory effects [75,76,77].

*C. racemosa* contained more pigments than *U. fasciata*. However, the two species demonstrated a high content of pigments and carotene, especially for *C. racemosa*. Although the amount of β-carotene for *C. racemosa* is low compared to chlorophyll a and b in the present study, it is still more than 40 times higher than that found in the study by Paul et al. [64] on the same species of *C. racemosa*. The amount of β-carotene previously reported for *Ulva* sp. (0.031% of dw) was much lower than that found for the present *U. fasciata* [54]. According to Paul et al. [64], the variation in the amount of pigments is comparable to fatty acids, as both are greatly affected by season and geographic location. Furthermore, β-carotene and other pigments in *C. racemosa* and *U. fasciata* vary as they are affected by time, season, cell growth, or plant location. Chlorophylls may also be used in human health; they can prevent cancer and have positive effects on inflammation and wound healing [54]. Studies carried out by Hsu et al. [78] have shown that chlorophylls directly act as reducers of free radicals and have the potential to protect lymphocytes against oxidative DNA damage by H_2_O_2_. Moreover, natural chlorophylls prevent lipid peroxidation of low-density lipoprotein (LDL). The second most important pigment is carotenoid, which is crucial due to its nutraceutical and antioxidative properties, especially in preventing pathologies caused by oxidative stress [79]. β-Carotene has anticancer properties and is absorbed ten times more easily by the body than its synthetic version [80]. Pigments from macroalgae are also important in the health food industry [19]. 

The sulfated cell-wall constituents may be classified into two of the three groups originally designated by Percival [81]: (a) sulfated xyloarabinogalactans and (b) sulfated glucuronoxylorhamnans and glucuronoxylorhamnogalactans. The sulfated xyloarabinogalactan group of cell-wall polysaccharides is present in *Caulerpa* [69,82]. Water-soluble heteropolysaccharides derived from *Caulerpa racemosa* are described as branched sulfated polymers containing 3-linked galactose, terminal- and 4-linked xylose, and as 4- and 3,4-linked arabinose residues [32,69]. The sugar composition of the *C. racemosa* extract (HWE) in the present study is somewhat similar to the results of Ghosh et al. [32], who used the same method of extraction from *C. racemosa* collected in Western India and showed the presence of glucose, xylose, and galactose; smaller amounts of mannose; and traces of rhamnose, arabinose, and fucose. We obtained high percentages of glucose and galactose in HWE, which is in line with Ghosh et al. [32], and other dominant sugars such as mannose and xylose were also present. The ulvan backbone is most commonly composed of α- and β-(1,4)-linked monosaccharides (rhamnose, xylose, glucuronic acids, and iduronic acids) with characteristic repeating disaccharide units [83]. Ulvans from *U. armoricana* [84] and *U. rotundata* [36] demonstrated that they consisted of rhamnose, glucose, xylose, glucuronic acids, and iduronic acids. These results showed that rhamnose was the dominant sugar, followed by sulfates and glucose. These sugars also indicate the presence of ulvans in the extracts. According to Lahaye and Robic [84], alcohol extraction decreased the content of minor sugars and glucose in the material. These small proportions of total sugar composition in all fractions in the present study could be attributed to the method of extraction used. The presence of glucose and other sugars in the present study indicates the presence of other types of saccharides besides ulvans. According to Lahaye and Robic [84], these other sugars are probably not in the form of free oligosaccharides since they were not removed during dialysis. 

The IR spectrum of HWE confirmed the presence of sulfate ester, sugars, and proteins. Some *C. racemosa* extracts reported the presence of N-H involved in metal adsorption binding processes [30,85]. This result is also similar in the study of Ghosh et al. [32]. The Si-C band can be attributed to observed silica particles or those attached to the thallus during collection, even after cleaning and drying of samples. A similar peak was reported in Sarada et al. [30] for *Caulerpa fastigiata*, where the presence of silica from diatomaceous earth in algal waste and composite material was determined. The asymmetrical stretching band attributed to the presence of OH groups, which is within the spectral bands of the uronic acids, was reported in a previous study for *Ulva lactuca* polysaccharide fractions [34]. These similar intensity bands were also found in the previous study for *Ulva armoricana* and *Ulva rotundata* fractions [36]. The presence of sulfate groups was also confirmed in the ulvan fraction by S=O elongation, C-O-S elongation in the axial position, and C-O-S deformation in the equatorial position. According to Olasehinde et al. [34], the presence of sulfate groups in algal polysaccharides are correlated to their biological activities or are synthesized in the algal Golgi bodies. These bands are due to these sugar cycles, according to Robic et al. [36], and are a typical signal for ulvans [34,35,36,86]. The FTIR spectrum of *U. fasciata* crude ulvan extract confirmed the presence of sulfate groups and sugars. The presence, degree, and distribution of the sulfate groups are important in determining the biological activity of ulvan [86].

Viral infections count as the most predominant cause of death in humans worldwide. Marine seaweeds have proven to be a rich source for active antiviral metabolites. Polysaccharides extracted from seaweeds exhibit antiviral activity against a wide spectrum of viruses, including major human pathogenic agents such as human immunodeficiency virus (HIV), herpes simplex virus (HSV), vesicular stomatitis virus (VSV), and cytomegalovirus (CMV) [87,88,89]. Among the different deadly viruses, *Herpesviridae*, a large family, is responsible for a wide range of mild to severe infections in humans. The most notable herpes viruses belonging to Alphaherpesvirinae are herpes simplex virus type 1 (HSV-1) and herpes simplex virus type 2 (HSV-2), which have been widely studied. Typically, HSV-1 is associated frequently with orofacial infections and encephalitis. These viruses can establish persistent, long-term, latent infections in sensory neurons and cause lesions at the entry point of the human body. There is no definitive vaccine as yet against numerous prevalent viral infections, including herpes simplex viruses (HSV-1 and HSV-2). Additionally, these characteristic features of latency enhance the pathogenicity of HSV [89]. Kidgell et al. [83] reported that the antiviral activity of ulvan extends to different enveloped viruses of HSV, Newcastle disease, dengue, yellow fever, influenza, and measles virus [83,90]. In this study, the ulvan shows no activity in the concentration range tested (1 to 200 μg/mL). However, according to the methodology used to evaluate the antiviral effects of ulvans against viral targets, the concentrations of ulvan required to inhibit viral yield by 50% are mixed, ranging from weak (IC_50_ > 150 μg/mL) [91,92] to significant (IC_50_ = 0.1–30 μg/mL) [93,94,95]. Variations in the antiviral activity of ulvan from different sources indicate a significant effect of structure. However, there are only a few studies that probe the antiviral activity of ulvan and, consequently, the understanding of its structure–activity relationship is restricted [83].

The hot water extract from *C. racemosa* showed antiherpetic activity, with an EC_50_ of 85.59 ± 26.27 µg/mL. The antiherpetic activity of HWE had previously been demonstrated by Ghosh et al. [32]. The hot water sulfated polysaccharide fraction isolated from *Caulerpa racemosa* collected in India was considered a selective inhibitor of thymidine kinase (TK) acyclovir-resistant HSV-1 strains in Vero cells with an EC_50_ of approximately 2.2–4.2 µg/mL [32]. The HWE studied by Ghosh et al. [32] appears to be more effective. The difference with antiherpetic activity can be explained not only by the monosaccharide composition, sulfate content, and geographical positions (India/Philippines), but also by the sensitivity of the methods used. While the extraction process is comparable, the HWE was evaluated for cytotoxicity on Vero cells using the MTT method, and the antiherpetic activity of HWE was determined by a plaque reduction assay against reference strains of HSV. Wang et al. [88] reported that sulfated polysaccharides from *Caulerpa brachypus* had antiviral activity against HSV-1 (EC_50_ = 9.6 µg/mL). The authors showed that antiviral activity has a direct correlation with the degree of sulfation and the polysaccharide sugar units found (rhamnose, xylose, and glucose).

Viral infections are often caused by oxidative processes, favoring replication in infected cells, induction, and inhibition of cell proliferation [96]. In patients affected by herpes simplex, it was observed that an increase of the reactive oxygen species (ROS)-induced membrane phospholipid peroxidation caused dysfunction of vital cellular processes such as membrane transport and mitochondrial respiration. Antioxidants are effective in protecting living organisms against oxidative damages caused by ROS. Discovery and screening of antioxidant compounds with antiviral properties are promising since the treatment of viral diseases requires the suppression of viral replication and the promotion of cell survival. Hot water extract from *C. racemosa* showed antiviral and antioxidant activities. Several polysaccharides extracted from *Caulerpa* have shown antioxidant activities: the *C. lentillifera* polysaccharide produced by ultrasonic-assisted extraction presented good radical scavenging activities against the DPPH radical [97]. The *C. lentillifera* polysaccharide antioxidant activity might be influenced by sulfate and uronic acid contents [97]. Fernando et al. [33] reported DPPH activity, with an IC_50_ of >2 mg/mL, of a polysaccharide extracted from *C. racemosa*. The authors also suggested the influence of sulfate and polyphenol content. This sulfated polysaccharide had galactans and mannans from its major sugars (15.36–32.71% of dw). Galactans were also present in the *C. racemosa* fraction in the present study, which is in agreement with Fernando et al. [33]. 

The *Caulerpa racemosa* hot water extract showed great potential in human health development. Antiviral activities of antioxidants acting against viral infection can be exploited. However, various factors mentioned must be understood better; therefore, extracts from *Caulerpa racemosa* must be studied further with the investigation of the structure of sulfated polysaccharides and the determination antiviral and antioxidant mechanisms of action representing top priorities.

## 4. Materials and Methods

*Caulerpa racemosa* var. Forsskål and *Ulva fasciata* Delile were collected on August 15–16, 2017 (rainy season) along the coastal area of Barangay Lawigan (8.226° N and 126.431° E), Bislig City, Surigao Del Sur, Philippines. The area has a 34‰ salinity, a temperature of 27 °C, and a pH of 8.16. Algae were immediately rinsed thoroughly with running fresh water to remove the remaining sand and epiphytes and left to dry under the shade, avoiding sunlight for 1 to 2 weeks. The dried samples (about 1 kg each) were then put in zip locks with corresponding labels (scientific name, date, place of collection, and other information pertinent to the seaweed) and finally transported to the LBCM in Vannes, France, where they were ground or cut into small pieces, freeze-dried, and stored in the dark for further analysis.

### 4.1. Biochemical Composition 

Protein, uronic acid, neutral sugar, sulfate group, and polyphenol contents: The biochemical composition of *C. racemosa* and *U. fasciata* were defined as percentages of each compound found in the total dry weight of raw material. All analyses were performed in triplicate. To characterize the seaweed, 10 mg freeze-dried matter was mixed with 5 mL of 1 M HCl in a sealed vial at 100 °C for 2 h, after which 5 mL of 1 M NaOH was added. The final solution was used to measure the protein, uronic acid, neutral sugar, and polyphenol contents. Neutral sugars were determined by the phenol–sulfuric acid colorimetric method, as described by Dubois [98], using anhydrous D-glucose (0–100 μg/mL) as the standard. The uronic acid content was quantified using the meta-hydroxydiphenyl (MHDP) method [99], with glucuronic acid as the standard (0–100 μg/mL). The bicinchoninic acid colorimetric (BCA) method [100] with a Micro BCA assay kit (ref. 23228, Thermo Scientific, Interchim, Montluçon, France) was used to measure the protein content. For the protein estimation, bovine serum albumin (0–100 μg/mL) was used as the standard. Polyphenols were quantified using the Folin–Ciocalteu method [101], using gallic acid as the reference. Water extraction was performed to measure the free sulfate group using the Azure A method [102]. For the extraction, 10 mg freeze-dried seaweed was mixed with 5 mL of ultrapure water in a sealed vial at 100 °C for 2 h, after which 5 mL of ultrapure water was added. For this estimation, sulfated dextran (17%) (0–100 μg/mL) was used as the standard. Total ash was determined gravimetrically after the incineration of samples, followed by 2 h at 585 °C.

### 4.2. Lipids and Fatty Acids 

Lipid extraction was performed following the method of Kendel et al. [103]. Lipid content was determined gravimetrically, and fatty acids were converted to fatty acid methyl ester (FAME). Finally, samples containing FAME were analyzed using a gas chromatography system coupled with flame ionization detection (GC-FID, TRACE 1300, Thermo-Fischer Scientific, Milan, Italy) and equipped with a capillary column CP-Sil 5 CB (60 m × 0.25 mm × 0.25 µm); the carrier gas was nitrogen (0.5 mL/min). The injector and detector were set at 250 °C, and a temperature gradient was used for FAME analysis: the temperature was held at 170 °C for 4 min and programmed to 300 °C at 4 °C/min. The FAs were identified by comparing the retention time with that of a commercial mixture (Supelco 37 Component FAME Mix, ThermoScientific, Illkirch, France). The relative proportions of the individual acids were calculated by the ratio of their peak area and expressed as percentages of total fatty acids.

### 4.3. Nutritional Quality Indexes

The nutritional quality indexes were assessed according to different parameters calculated with the concentration of fatty acids. For all calculations needing a distinction between C18:3ω6 and ω3 and C20:3ω6 and ω3, the calculations were done in two ways. The first one considered all the percentages as ω6, and the second one considered all the percentages as ω3.

(1) C18/C20 PUFA Ratio:

(1) $$\frac{C18}{C20}=\frac{\sum^{\;}C18\mathrm{PUFA}}{\sum^{\;}C20\mathrm{PUFA}}$$

(2) ω6/ω3 Ratio:

(2) $$\omega 6/\omega 3=\frac{\sum^{\;}\mathrm{PUFA}\omega 6}{\sum^{\;}\mathrm{PUFA}\omega 3}$$

(3) Atherogenicity Index (AI) [26]:

(3) $$\mathrm{AI}=\frac{C12:0+4\times C14:0+C16:0}{\sum^{\;}\mathrm{MUFA}+\sum^{\;}\mathrm{PUFA}\omega 6+\sum^{\;}\mathrm{PUFA}\omega 3}$$

(4) Thrombogenicity Index (TI):

(4) $$\mathrm{TI}=\frac{C14:0+C16:0+C18:0}{0.5\times \sum^{\;}\mathrm{MUFA}+0.5\times \sum^{\;}\mathrm{PUFA}\omega 6+3\times \sum^{\;}\mathrm{PUFA}\omega 3+\frac{\sum^{\;}\mathrm{PUFA}\omega 3}{\sum^{\;}\mathrm{PUFA}\omega 6}}$$

(5) Hypocholesterolemic/Hypercholesterolemic (h/H) Fatty Acids:

(5) $$h/H=\frac{C18:1\omega 9+C18:2\omega 6+C20:4\omega 6+C18:3\omega 3+C20:5\omega 3}{C14:0+C16:0}$$

(6) PUFAs/SFAs:

(6) $$\mathrm{PUFA}/\mathrm{SFA}=\frac{\sum^{\;}\mathrm{PUFA}}{\sum^{\;}\mathrm{SFA}}$$

where PUFAs represents polyunsaturated fatty acids, MUFAs represents monounsaturated fatty acids, and SFA represents saturated fatty acids.

Unsaturation index (UI) was calculated by multiplying the number of double bonds by the percentage of each fatty acid, followed by summing up their contributions [104].

### 4.4. Cell Wall Polysaccharides

Ground freeze-dried samples were extracted sequentially with 96% ethanol, chloroform/methanol (1:1, *v*/*v*), and acetone in a Soxhlet apparatus before polysaccharide extraction. The depigmented samples (4 g) from *C. racemosa* were extracted following the method of Ghosh et al. [32] with distilled water (200 mL, pH 6.5) at 80 °C for 30 min, three times (hot water extracted (HWE)); extracts were dialyzed extensively against distilled water using a membrane having molecular weight cutoff of 12 kDa. Soluble materials were recovered by diluting the retentate with distilled water and ethanol (1:5; *v*/*v*). *U. fasciata* polysaccharide extraction was based on the work of Robic et al. [36] and Hardouin et al. [105]. Depigmented samples (10 g) were also extracted sequentially: (1) samples were extracted distilled water (200 mL) by maceration at 90 °C for 2 h and were filtered on Buchner cloth to obtain the aqueous fraction; (2) the crude extract of ulvans was obtained by ethanol precipitation (1:5, *v*/*v*) of the aqueous fraction for 24 h at 4 °C; and (3) the precipitate obtained (raw ulvan) was filtered, dried, and stored at −20 °C.

#### 4.4.1. Monosaccharide Analysis of Polysaccharides

The simple sugar composition of the samples was determined by high-performance anion-exchange chromatography (HPAEC, Sunnyvale, CA, USA) with pulsed amperometric detection (PAD) (Thermo Dionex, city, France), based on the procedure of Pliego-Cortés et al. [106] with brief modifications. HWE from *C. racemosa* and crude ulvan from *U. fasciata* (4 mg dw each) were subjected to acid hydrolysis for 48 h at 100 °C using 110 μL of 1 M HCl and 1 mL Milli-Q water in a flame-sealed glass ampule. The mixture was neutralized with 110 μL of 1 M NaOH and 780 μL of Milli-Q water containing deoxyribose (internal standard) to a final concentration of 50 ppm. All samples were filtered with a 0.22 μm filter paper. Details of the elution program can be found in Pliego-Cortés et al. [106]. Monosaccharides were identified and quantified based on their standard curves at different concentrations (1.95–125 ppm), including fucose, rhamnose, arabinose, glucosamine, galactose, glucose, mannose, xylose, fructose, ribose, glucoheptose, and glucuronic acid. Results were expressed as micrograms of monosaccharides per milligram of dry weight (μg mg^−1^ dw).

#### 4.4.2. Fourier-Transform Infrared (FTIR) Spectroscopy 

Spectra of HWE from *C. racemosa* and crude ulvan from *U. fasciata* were determined using a Nicolet iS 5 FTIR spectrometer (Thermo Scientific, Madison, WI, USA) with a diamond crystal plate. The spectra were recorded in reflexing mode from 4000 to 500 cm^−1^ as the percentage transmittance with 16 scans of the samples. A background scan with the diamond plate in place was run before each analysis. The detected peaks after Fourier transformation were then compared with algal polysaccharide standards.

### 4.5. Amino Acid Composition

The amino acid composition of the two species was determined separately by GC-FID after liquid phase hydrolysis of the samples with 6 M HCl at 110 °C for 24 h. The amino acid samples were prepared according to the procedure recommended by the EZfaast (Phenomenex, Torrance, CA, USA) method, consisting of a solid-phase extraction step followed by derivatization and a liquid/liquid extraction. Derivatized amino acids were analyzed by gas chromatography coupled to a flame ionization detector (GC-FID, TRACE 1300, Thermo-Fischer Scientific, Milan, Italy), using a Zebron ZB-AAA-GC column (10 m × 0.25 mm, Agilent, CA, USA). Two-microliter samples were introduced into the injector (250 °C) with a split ratio of 1:10 and separated using the following program: 110 °C to 320 °C (an increase of 32 °C/min), held for 2 min. Nitrogen was used as the carrier gas at a constant flow of 1.7 mL/min, and the detector was set at 320 °C. The signals were recorded using Chromeleon 7.2 software (Dionex, Thermo-Fischer Scientific, Sunnyvale, CA, USA). Amino acids were identified by their time of retention and quantified by their response factor relative to Norvaline, the internal standard added at a concentration of 200 μmol/L.

### 4.6. Mineral Analysis by Flame Atomic Absorption Spectrophotometry

Major mineral elements (Ca, Mg, Na, and K) and trace elements (Fe, Zn, Cu, and Mn) were determined using a PerkinElmer Analyst 200 atomic absorption spectrophotometer (AAS) with a single hollow cathode lamp for each element and an air-acetylene burner. The 200 mg raw samples of dried seaweed were placed into digestion vessels with 1.3 mL of 1 M HCl, and 19.7 mL of ultrapure water was added to make the final sample concentration of 10 mg/ML. This was later incubated for 48 h at 118 °C. Samples were filtered (0.22 μm, Minisart High Flow; Sartorius Stedim Biotech, Göttingen, Germany) before analysis. Filtered samples were diluted with distilled water to obtain a 1 mg/mL concentration. Quantification was performed using standard ranges depending on the element.

### 4.7. Pigment Analysis

Pigment analysis was based on the method used by Silkina et al. [39] with some modifications. Pigment extract from raw material (20 mg) was diluted with methanol (2 mL, at 4 °C) and sonicated (pulse 2, amplitude 100) for 2 min in test tubes. Samples were kept in the darkroom (4 °C, 24 h), centrifuged (36,000× *g*, 4 min), and then filtered (0.22 μm) before analysis. The analysis was performed by high-performance liquid chromatography (HPLC) on a Nucleodur C18 column (EC 250/4.6 100–5 C18ec Macherey-Nagel). A two-solvent elution (eluent A 100% 0–27 min, eluent B 100% 27–42 min) was used at a flow rate of 1.4 mL/min to separate the pigments (eluent A, methanol/acetonitrile/ammonium acetate 2.3 M in water (51/36/13, *v*/*v*/*v*); eluent B, ethyl acetate/acetonitrile (7/3, *v*/*v*)). Pigments were detected and characterized by diode array measurements of their absorption spectra recorded between 300 and 600 nm. Chromatograms were registered at 440 nm, and standards of chlorophyll *a* and *b* and β-carotene (Sigma–Aldrich) were used for pigment identification and quantification. Pigment concentrations were expressed as mg/g.

### 4.8. Biological Activities

#### 4.8.1. Cytotoxicity and Antiviral Activity of Polysaccharides Evaluated by Cell Viability

Vero cell lines (line No. ATCC CCL81) were cultivated in Eagle’s MEM supplemented with 8% fetal calf serum (FCS, Eurobio, France); 1% l-glutamine (200 mM); and 1% PCS (penicillin (10,000 U), colimycin (25,000 U), and streptomycin (10 mg)). The culture was performed at 37 °C under a 5% CO_2_ atmosphere, and the medium was renewed daily. Herpes simplex virus type 1 (HSV-1; family *Herpesviridae*) was provided by Pr. Agut (Laboratoire de Dynamique, Epidémiologie et Traitement des Infections Virales de la Pitié Salpêtrière Paris, France). Dilutions of samples (1 μg/mL to 200 μg/mL) were prepared in Eagle’s MEM supplemented with 8% FCS and distributed into a 96-well plate. One hundred microliters of cellular suspension (3.5 × 10^5^ Vero mammalian cells/mL) in supplemented Eagle’s MEM was added to each well. Cells were infected by the HSV-1 at a multiplicity of infection of 0.001 ID_50_/cells. The 96-well plate was incubated for three days at 37 °C with 5% CO_2_. Cytotoxicity was tested using cell viability by the neutral red dye method. Optical density (OD) was measured at 540 nm. The 50% cytotoxic concentration (CC_50_) was defined as the concentration of seaweed extract that reduced the OD of treated cells to 50% of that of untreated cells. The antiherpetic compound acyclovir was used as a reference inhibitor. The 50% effective antiviral concentration (EC_50_) was expressed as the concentration that achieved 50% protection of virus-infected cells [107].

#### 4.8.2. Antioxidant Activity

The radical scavenging activity of antioxidant 2,2-diphenyl-1-picrylhydrazyl (DPPH) was determined according to Terme et al. [55]. Solutions of butylated hydroxyanisole (BHA) and butylated hydroxytoluene (BHT) at different concentrations ranging from 1 to 20 µg/mL in methanol (final concentration) were tested as a positive control. Sample solutions of *C. racemosa* and *U. fasciata* HWE and crude ulvan were prepared in different concentrations by diluting the stock solution in water (0–5000 μg/mL). IC_50_ (corresponding to the concentration sufficient to obtain 50% of a maximum scavenging capacity) of samples was determined based on the regression obtained from the dose–response curve.

## 5. Conclusions

The species studied presented different nutritional values based on their nutritional characteristics, while presenting different biological activities. Wild *Caulerpa racemosa* collected in the Philippines offers real nutritional value based on the results of our studies. The biochemical components found in *Ulva fasciata* that have the potential for nutraceutical application were palmitic acid and PUFAs (C20:4ω6 and C20:5ω3). Results obtained from this study suggested that *Ulva fasciata* could still be utilized as a functional ingredient in the food and health industries because of its considerable amount of C22:1ω9 (erucic) and C24:1ω9 (nervonic) acids, which are reported to prevent demyelinating disease. In the specific example of antioxidants, our results suggest that fresh seaweed should be preferred for human consumption.

## Figures and Tables

**Table 1:** Biochemical composition (% of dry weight) of raw and purified fractions from *Caulerpa racemosa* and ulvan from *Ulva fasciata*.

	Content (% Dry Algal Material)							
Sample	Yield	Proteins	Lipids	Uronic Acids	Sulfate Groups	Neutral Sugars	Polyphenols	Ash
***C. racemosa***								
Raw		19.9 ± 0.5	4.5	3.6 ± 0.4	13.0 ± 1.5	31.2 ± 4.9	4.9 ± 1.6	29.4 ± 0.9
HWE	13.0	8.8 ± 0.9	/	1.6 ± 0.4	10.5 ± 0.3	16.6 ± 1.7	2.4 ± 0.9	/
***U. fasciata***								
Raw		11.1 ± 0.7	4.1	20.7 ± 4.8	25.4 ± 0.3	25.0 ± 3.5	5.8 ± 0.8	23.3 ± 2.2
Crude ulvan	23.71	8.0 ± 1.2	/	10.0 ± 0.2	3.12 ± 0.1	9.5 ± 1.3	5.0 ± 1.2	/

Values are means ± SD (*n* = 9) except for lipids. / = not determined.

**Table 2:** Fatty acids (% of total fatty acids (TFAs)) of *Caulerpa racemosa* and *Ulva fasciata*.

Fatty Acid	*C. racemosa* (%)	*U. fasciata* (%)
C4:0 (Butyric)	5.3 ± 0.3	0.8 ± 0.0
C6:0 (Caproic)	0.2 ± 0.0	/
C11:0 (Undecanoic)	0.1 ± 0.1	/
C12:0 (Lauric)	1.2 ± 0.0	0.1 ± 0.0
C13:0 (Tridecanoic)	0.3 ± 0.0	/
C14:0 (Myristic)	0.6 ± 0.1	1.7 ± 0.0
C15:0 (Pentadecanoic)	0.4 ± 0.0	0.2 ± 0.0
C16:0 (Palmitic)	27.6 ± 0.6	59.9 ± 0.5
C17:0 (Heptadecanoic)	0.3 ± 0.2	0.2 ± 0.0
C18:0 (Stearic)	3.3 ± 0.0	3.2 ± 0.0
C20:0 (Arachidic)	0.4 ± 0.0	0.2 ± 0.0
C21:0 (Heneicosanoic)	0.1 ± 0.0	0.2 ± 0.0
C22:0 (Behenic)	1.00 ± 0.1	0.9 ± 0.0
C23:0 (Tricosanoic)	0.3 ± 0.0	nd
C24:0 (Lignoceric)	0.1 ± 0.0	4.6 ± 0.1
Total SFAs	41.3 ± 0.4	72.1 ± 0.4
C15:1	0.5 ± 0.0	/
C16:1 (Palmitoleic)	4.5 ± 0.1	3.0 ± 0.0
C17:1	1.0 ± 0.5	0.1 ± 0.0
C18:1ω9 (Oleic)	28.1 ± 0.0	5.9 ± 0.0
C20:1ω9 (*cis*-11-Eicosenoic)	0.4 ± 0.5	0.7 ± 0.0
C22:1ω9 (Erucic)	nd	1.7 ± 0.5
C24:1ω9 (Nervonic)	0.2 ± 0.0	1.2 ± 0.0
Total MUFAs	34.6 ± 0.7	12.7 ± 0.5
C18:2ω6 (Linoleic)	9.0 ± 0.1	5.3 ± 0.0
C18:3ω6 & ω3 (γ & α-Linolenic)	7.2 ± 0.1	4.0 ± 0.0
C20:2ω6 (*cis*-Eicosadienoic)	0.6 ± 0.0	0.4 ± 0.0
C20:3ω6 & ω3 (*cis*-Eicosatrienoic)	1.2 ± 0.0	0.3 ± 0.0
C20:4ω6 (Arachidonic)	0.7 ± 0.0	0.4 ± 0.0
C20:5ω3 (*cis*-Eicosapentaenoic)	0.7 ± 0.6	/
C22:2ω6 (*cis*-Docosadienoic)	0.4 ± 0.0	nd
C22:6ω3 (*cis*-Docosahexanoic)	1.8 ± 0.0	nd
Total PUFAs	21.6 ± 0.2	10.5 ± 0.0
Unidentified	2.5	4.7

Average reference values (% TFAs ± SD); TFAs = total fatty acids; SD = standard deviation; nd = not detected; / = detected at less than 0.1%. SFAs = saturated fatty acids; MUFAs = monounsaturated fatty acids; PUFAs = polyunsaturated fatty acids. C18:3ω6 and ω3 and C20:3ω6 and ω3 were found merged during GC analysis.

**Table 3:** Nutritional quality indexes of fatty acids from *Caulerpa racemosa* and *Ulva fasciata*.

Seaweed	C18/C20	ω6/ω3	AI	TI	h/H	PUFA/SFA	UI
*C. racemosa*	4.99	0.97–7.54	0.56	0.56–0.91	1.36–1.62	0.52	97
*U. fasciata*	8.03	1.39–120.40	2.89	2.78–5.48	0.19–0.25	0.15	39

AI = atherogenic index; TI = thrombogenic index; h/H = fatty acid hypocholesterolemic/hypercholesterolemic ratio; PUFA = polyunsaturated fatty acid; SFA = saturated fatty acid; UI = unsaturation index.

**Table 4:** Monosaccharide composition as a percentage of total sugars and total content of sugars (μg mg^−1^ dw) in hot water extract (HWE) from *Caulerpa racemosa* and crude ulvan from *Ulva fasciata*.

	*C. racemosa* HWE	*U. fasciata* Crude Ulvan
Monosaccharide	% of Total Sugars	
Fucose	1.30 ± 0.05	/
Rhamnose	1.07 ± 0.05	53.98 ± 1.5
Arabinose	0.29 ± 0.01	/
Glucosamine	0.95 ± 0.04	/
Galactose	15.62 ± 0.3	0.68 ± 0.01
Glucose	52.42 ± 1.3	26.64 ± 0.7
Mannose	12.61 ± 0.1	/
Xylose	8.21 ± 0.1	7.85 ± 0.2
Fructose	4.09 ± 0.7	/
Glucoheptose	0.26 ± 0.01	0.51 ± 0.02
Glucuronic acid	1.43 ± 0.02	1.38 ± 0.03
Others	1.75 ± 0.07	8.96 ± 2.5
Total (μg mg^−1^ dw)	198.13 ± 8.23	62.27 ± 1.61

Others represents the sum of non-identified monosaccharides; / = not detected. Data are means ± SD (*n* = 2). SD = standard deviation.

**Table 5:** Amino acid compositions of *Caulerpa racemosa* and *Ulva fasciata* expressed as a percentage (%) of the total amino acids detected by gas chromatography.

		*C. racemosa* (% of Total AAs)	*U. fasciata* (% of Total AAs)	Ovalbumin ^a^ (% of Total AAs)
Essential Amino Acids (EAAs)	Threonine	4.27 ± 0.10	3.12 ± 0.13	3.95
	Valine	8.63 ± 0.02	8.50 ± 0.07	7.10
	Lysine	5.96 ± 0.14	7.92 ± 0.44	10.14
	Isoleucine	6.14 ± 0.10	4.98 ± 0.17	6.32
	Leucine	9.64 ± 0.09	9.31 ± 0.28	8.16
	Phenylalanine	5.79 ± 0.07	6.07 ± 0.35	5.39
	Histidine	2.41 ± 0.00	1.41 ± 0.08	5.39
	Methionine	2.37 ± 0.07	0.86 ± 0.20	4.08
	Total EAAs	45.28 ± 0.12	42.17 ± 1.31	50.52
Non-Essential Amino Acids (NEAAs)	Aspartic acid	11.53 ± 0.11	10.95 ± 0.46	8.16
	Serine	3.54 ± 0.07	3.11 ± 0.46	8.95
	Glutamic acid	18.70 ± 0.17	15.49 ± 0.14	13.03
	Glycine	5.66 ± 0.04	6.87 ± 0.16	4.47
	Alanine	7.42 ± 0.12	10.85 ± 0.52	8.82
	Tyrosine	3.09 ± 0.14	4.04 ± 0.08	2.37
	Proline	4.39 ± 0.07	5.13 ± 0.07	3.69
	Hydroxyproline	0.39 ± 0.05	1.39 ± 0.00	ND
	Total NEAAs	54.72 ± 0.12	57.83 ± 1.31	49.48
	EAA/NEAA ratio	0.83 ± 0.00	0.73 ± 0.04	1.02

Values presented for *C. racemosa* and *U fasciata* are means of duplicates ± SD. ^a^ Values for the ovalbumin composition in amino acids were adapted from Kazir et al. [38].

**Table 6:** Composition of macroelements and microelements (% of algal dw), recommended daily intakes, and recommended daily allowances of *C. racemosa* and *U. fasciata*.

Species	Macroelements					Microelements			
	Na	K	Ca	Mg	Na/K	Fe	Zn	Mn	Cu
RDI ^a^ (g/day)	2.0	3.8	0.84	0.350		0.006	0.012	0.0055	0.0017
Upper limit intake ^b^ (g/day)	2.3	/	2.5	0.35		0.045	0.040	/	0.01
*C. racemosa*	7.48 ± 0.00	5.30 ± 0.00	3.55 ± 0.02	0.50 ± 0.0	1.41	0.49 ± 0.02	0.42 ± 0.02	0.006 ± 0.0	0.11 ± 0.0
% RDI	29.9	11.2	33.8	11.4		653.3	280.0	8.7	517.6
RDA (g/day)	30.7	/	70.4	70.0		9.2	9.5	/	9.1
*U. fasciata*	4.71 ± 0.00	1.51 ± 0.00	4.80 ± 0.03	2.24 ± 0.00	3.12	1.77 ± 0.01	0.27 ± 0.01	0.003 ± 0.0	0.06 ± 0.0
% RDI	18.8	3.2	45.7	51.2		2360	180	4.4	282.3
RDA (g/day)	48.8	/	52.1	78.4		2.5	14.8	/	16.7

^a^ RDI = Recommended daily intake and upper limit intake values for a male adult with a bodyweight of 70 kg according to the World Health Organization (2002); ^b^ Upper limit intake according to National Health and Medical Research Council (2006); % RDI is based on a daily intake of 8 g dry weight of seaweed; RDA = recommended daily allowance. Data are means ± SD (*n* = 2). SD = standard deviation.

**Table 7:** Pigment contents (mg/g dw) of *Caulerpa racemosa* and *Ulva fasciata*.

Seaweeds	Chlorophyll *a*	Chlorophyll *b*	β-Carotene	Total Chlorophyll	Total Pigments
*C. racemosa*	42.15 ± 0.21	81.42 ± 0.24	17.26 ± 1.88	123.58	140.84
*U. fasciata*	2.18 ± 0.74	4.64 ± 0.60	0.72 ± 0.00	6.82	7.54

Data are means ± SD (*n* = 2). SD = standard deviation.

**Table 8:** Evaluation of cytotoxicity and antiviral and antioxidant activities of polysaccharide fractions.

Samples	CC_50_ (µg/mL)	EC_50_ (µg/mL)	IC_50_ (µg/mL)
HWE	>200.00	85.59 ± 26.27	276.08 ± 52.62
Zovirax	>200.00	0.47 ± 0.07	
Crude ulvan	>200.00	>200.00	4482.49 ± 635.05
Zovirax	>200.00	0.37 ± 0.03	
BHA			7.05 ± 0.59
BHT			5.32 ± 0.59

The 50% cytotoxic concentration (CC_50_) is the concentration that reduced the absorbance of mock-infected cells to 50% of that of controls. The 50% antiviral effective concentration (EC_50_) is the concentration that achieved 50% protection of virus-infected cells from the HSV-induced destruction. The 50% inhibitory concentration (IC_50_) is the concentration sufficient to obtain 50% of a maximum scavenging capacity. BHA = butylated hydroxyanisole; BHT = butylated hydroxytoluene.
